# Transverse Fracture of the Patella

**Published:** 1887-08

**Authors:** J. Y. Brown

**Affiliations:** Henderson, Ky.


### TRANSVERSE FRACTURE OF THE PATELLA.

BY J. Y. BROWN, M. D., HENDERSON, KY.

[Read before the Mississippi Valley Medical Society, in Annual Session at Crab Orchard Springs, July 12–15, 1887.]

I KNOW of no subject in the whole range of surgery which has been more thoroughly studied, both in regard to its pathology and treatment, than that of transverse fracture of the patella; and until very recently no satisfactory explanation has ever been offered to throw light upon the cause of non-osseous union which practically exists in nearly all cases of transverse fracture of this bone. From the birth of surgery to the present day, theory after theory has been advanced in explanation of this constantly existing result; and although each has seemingly been well sustained by anatomical, physiological and pathological facts, all alike have been disproven, and until within the last two years none could assign a cause and prove it. As has been the pathology, even worse has been the treatment; and but for the introduction of modern antiseptic surgery, it would have remained in that deplorable state, alike unsatisfactory to surgeon and patient, unscientific from the standpoint of modern investigations, and in some respects barbarous. As the views concerning both the pathology and treatment of this fracture have undergone so marked a change in the last two years, a separate consideration of the two subjects will, I trust, prove interesting to the society. I shall, therefore, first briefly consider its pathology from a modern standpoint, and afterwards the treatment.

An able article published in the March number, for 1887, of *The Annals of Surgery*, by Macewen of Glasgow, on "The Pathology of Transverse Fracture of the Patella," brings forward a most scientific and satisfactory explanation of the cause of non-osseous union in these cases; and in this he not only proves the correctness of his own views, but thoroughly disproves all pre-existing theories in regard to this matter. He opens his paper with an anatomical consideration of the knee-joint, laying especial stress on the point of insertion of the quadriceps extensor tendon and the anatomical distribution of the præpatella aponeurosis. In a series of anatomical dissections he illustrates the fact that the "tendon of quadriceps is for the most part inserted into the groove on the upper part of the patella, the strong aponeurosis being attached to its sides, while the ligamentum patellæ arises chiefly from its lower border." He shows that over the center of the anterior surface of the patellæ, continuous with the tendon of the quadriceps above and the ligamentum patellæ below, there exists a distinct band of aponeurotic tissue, completely covering the surface of the bone. Ten normal patellæ submitted to examination by Macewen showed the constancy of this aponeurosis; and I myself have made an examination of five cases in regard to this fact, and in each have found it as above described. Any one who will take the trouble to make this dissection can readily demonstrate to his thorough satisfaction both the point of insertion of the quadriceps tendon and the anatomical distribution of the præpatella structure, which play so important a part in the pathology of the fracture.

Now, it is well known that transverse fracture of the patella is in the majority of cases caused by violent muscular action, while in the attempt of maintaining an equilibrium during the act of falling. "The powerful force of the quadriceps causes the patella to be jerked upwards, relatively to the former, beyond the position where it usually rests, supported laterally by the femora-patella surface. The patella being held below by the power of the ligamentum patella, rests on the apex of its posterior vertical ridge, which thus becomes the point upon which is developed the greatest energy of the two opposing forces,—that of the contraction of the quadriceps on the one side, and the weight of the body on the other." When fracture occurs in the quadriceps tendon the upper fragment becomes tilted, so that the fracture surface presents anteriorly. Thus the shreds become entangled in the spicula of the fractured bone, and the soft parts are drawn into the interspace, by the flexing of the joint and the atmospheric pressure; hence, it is plain that with these structures between the fragments, together with the floor of the patella bursæ, approximation of the fragments is rendered impracticable. Consequently osseous union with this condition is impossible; fibrous union will be seriously modified. Now, the views of Doctor Macewen herein stated, are *not conclusions drawn from theoretical reasoning*, *but au facto* the fibrous aponeurosis,being more elastic than the bone, bridges over the interspace between the two fragments, and should the force be sufficently great, this aponeurosis ruptures and hangs in shreds of variable lengths which are forced into the hiatus between the fractured bone. When fracture occurs the præpatella structures,—viz., the fibrous aponeurotic tissue and floor of the patellæ bursæ become stretched and ruptured; and by reason of the flexed position of the joint, influence of atmospheric pressure, and the position of the upper fragment, these structures are forced into the hiatus and an irreducible separation is brought about. Prior to Macewen's investigation of this subject, there were three causes assigned, in explanation of non-osseous union in this fracture, all of which he considers and scientifically refutes. Although it may appear superfluous to consider here these theories—for truly the pathology as it now stands can but be clear and conclusive to the mind of every surgeon—still, it is a matter of some interest and may as well be considered.

In all works on surgery, non-osseous union has been attributed, first of all, to insufficient blood-supply to the patella, thereby causing a low vitality of the bone and rendering the out-pouring of ossific deposits, which enter prematurely into the repair of every fracture, poor and scanty. Now, this assumption is absolutely without foundation, for it has been shown by injected specimens that the blood-supply to this bone is not only abundant, but thoroughly distributed. Any one who has ever seen a joint opened in viewing this fracture, can bear evidence of this fact from the abundant parenchymatous hæmorrhage which ensues from the cancellated tissue of the fracture bone. Again in longitudinal fracture of this bone osseous union is the rule; and in rare cases of transverse fracture a like result follows; hence, were the above true, such a result would be impossible.

Not only is the parenchymatous hæmorrhage great in these cases, but, hæmorrhage is at times seen. Neither my collegues nor myself have been able to find mention of this fact in literature, but a case in point gives abundant proof. In a resection of the knee-joint made by Doctors Jenkins and Dixon after Volkman's method, and in which I was kindly invited to assist, the arterial hæmorrhage after the patella had been sawn through was so great that the Paquelin cautery had to be resorted to. How often this condition is present I am unable to say. Be this as it may, the fact still remains and is thoroughly substantiated, that the patella is abundantly supplied with blood, and the theory of insufficent blood-supply can be excluded from the hitherto presumably existing causes of non-osseous union.

Second, contraction of the quadriceps extensor tendon, which separates the fragment by pulling on the proximal one, may in some respects prevent union, if no attempt at reduction be made; but reduction is easy when properly practiced, consequently this alike with the first can be excluded.

Third, and lastly, the effusion of blood and serum into the joint, causing distension, and thereby separation, has been brought forward as a cause. The rapid absorption of this material renders its presence of little importance in the prevention of osseous union. Blood is a constant factor in every fracture, and forms, as is well known, one of the media of repair. Hence, by exclusion, we arrive at the true pathology of this fracture, and it only remains to adopt a proper method of treatment, based on these anatomical and pathological conclusions.

It is evident, if we accept Macewen's views of the pathology of this fracture, that none of the previous methods of treatment offer more than the prospect of an unsatisfactory result. Ligamentous union is all we can expect, and frequently we fail even in obtaining this, even granting that strong ligamentous union is gotten, partial or complete anchylosis is concomitant, and efforts at passive motion are accompanied by a stretching of the ligamentous band of union, thereby producing wide separation of the fragments and a necessary weakening and impaired function of the joint. On the contrary, should this more favorable union not occur, the condition is even worse, the fragments are then united by a thickening of the aponeurotic structures in front of the bone, and this condition—to quote Erichsen—always leaves a weakened limb and an unprotected joint. In consequence of the separation of the fragments and the folding of the bursæ, the fingers can be thrust in between the articular surfaces of the knee. It is true, anchylosis by manipulation and passive motion can be overcome, but it is accomplished by a stretching of the bond of union and a consequent separation of the fragments, so that even though mobility be completely restored, the joint is left in so weak a condition that it is relatively of little more value than one in an anchylosed state. This is no melancholy picture of the results to be expected from the ordinary methods of treatment. On this point all authorities agree, and doubtless there are many of you here present who can add a like experience to these. It would be useless to consider here all the manifold apparatus and devices which have been and are still being used in the treatment of these fractures

They are all adjusted with the same object; and, while they differ slightly in construction, the principle is the same; therefore what is said of one will apply alike to all.

In the repair of every fracture, osseous union is dependent primarily upon two conditions: First, it is necessary that the fragments be adjusted; and second, that they be maintained in position by some properly applied splint or bandage or by other means which I shall mention further on. Now, by any of the ordinary methods of treatment, neither of these conditions can be fulfilled, consequently the treatment promises little or nothing. After days of suffering and months of confinement the patient is left to lament a crippled condition and the pleasant anticipation of a big doctor's bill. The impossibility of getting osseous union by these methods is proven by results, as is the cause, by the pathology. It may be well to state here that I shall advocate in this paper the operation of opening the joint and wiring the fragments, contending that the idications and contra-indications are the same as those which govern us in the performance of any surgical operation, and that by this means only can we expect to get ideal results. I am well aware that on this point there is considerable diversity of opinion, but, after a careful examination of the results and mortality following, both lines of treatment, I am thoroughly satisfied that the operation of suturing the patella fragments, in the hands of a good man, is equally as safe, if not safer, than some of the former methods of treatment, and statistics show that results are incomparably better. As I before stated, all methods of treatment proposed for this fracture have for their object the accomplishment of two conditions, viz: Approximation of the fragments and the retention of them in position when approximated. Now, a glance at the pathology of this fracture will show that to accomplish this the joint must be opened, for unless the præpatella structures are removed from between the fragments approximation is impossible and osseous union is never gotten. All of you are more or less familiar with the usually recommended form of treatment laid down in most works on surgery. A consideration of them now would therefore be superfluous—all, with the exception of one, have this in their favor, namely, they are safe; and while they can accomplish comparatively little in the shape of good, they in no way endanger the life of the patient.

Before summing up the dangers and results to be expected from the two courses of treatment, I cannot refrain from making a few criticisms on the use of the malgaigne's hooks, a relic of barbarism which I trusted had long since been relegated to the tombs of antiquarian remains; but, to my surprise I find in the last edition of that scientific and ably edited journal, the *Annals of Surgery*, an article by Dr. Buchmore, Professor of Surgery in the Long Island College Hospital, in which he not only recommends them, but urges their use. Wherein there appears any advantages over other means of treatment, I fail to see; and certainly the tortures and dangers to which they subject a patient, should exclude them from the armamentarium of all scientific surgeons. Osseous union has never been gotten by this means of treatment, nor is it possible that such could result. Erysipelas necrosis and separation have been known to follow their use, and this, together with the pain in applying them and the absolute nothing in point of result which their application promises, is certainly sufficient ground to warrant a suit for malpractice, in case any complication should come up resulting from such treatment.

From this let us proceed to a general consideration of the two plans of treatment, viz.: That which consists of splints, hooks, bandages and the like, is that which consists in opening the joint and wiring the broken fragments. First, by adopting any of the former methods of treatment, what is the surgeon to expect? This question is easily answered; non-osseous union in every case; a weak joint; and in nearly all cases, partial or complete anchylosis. On the contrary, should the fragments be wired, a prognosis of rapid recovery; osseous union; and in almost every case, a complete restoration of the functions of the joint. I know there are many who contend that the operation is unsafe, but this statement cannot be substantiated. Has it not been proven that all inflammation is due to infection, and that, wherever pus is found, micro-organisms are present? Can we not say that by a thorough carrying out of the principles of antiseptic surgery this can be prevented, and by excluding infection, the operation is, comparatively speaking, dangerless. With the safeguard which antiseptics hold out against infection and the modern improvements in surgical technique, on surgeon can hesitate a moment in performing this operation. Let due care be taken in the thorough carying out of the antiseptic technique involved in the operation, and a favorable prognosis can be given in every case and the life of the patient is in no ways endangered. Should we hesitate in the performance of a surgical operation on the grounds of its being dangerous, and operate only on those cases devoid of danger, surgical science would dwindle down to the administration of drugs, for truly the simple exploration of a knee-joint is far more dangerous than wiring the patella when scientifically performed. No, the argument of danger can only be brought forward against the operation by those who are unfamiliar with and who disregard the teachings of antiseptic surgery. The operation in itself is an exceedingly simple one, success depending entirely on the antiseptic technique. It has been done any number of times, and I have been unable to find report ot any complication occurring, when due respect had been paid to the drainage and after-dressing.

